# Functional and evolutionary correlates of gene constellations in the *Drosophila melanogaster *genome that deviate from the stereotypical gene architecture

**DOI:** 10.1186/1471-2164-11-322

**Published:** 2010-05-24

**Authors:** Shuwei Li, Ching-Hua Shih, Michael H Kohn

**Affiliations:** 1 Department of Ecology and Evolutionary Biology, Rice University, 6100 Main Street, MS 170, Houston, Texas 77005, USA

## Abstract

**Background:**

The biological dimensions of genes are manifold. These include genomic properties, (e.g., X/autosomal linkage, recombination) and functional properties (e.g., expression level, tissue specificity). Multiple properties, each generally of subtle influence individually, may affect the evolution of genes or merely be (auto-)correlates. Results of multidimensional analyses may reveal the relative importance of these properties on the evolution of genes, and therefore help evaluate whether these properties should be considered during analyses. While numerous properties are now considered during studies, most work still assumes the stereotypical solitary gene as commonly depicted in textbooks. Here, we investigate the *Drosophila melanogaster *genome to determine whether deviations from the stereotypical gene architecture correlate with other properties of genes.

**Results:**

Deviations from the stereotypical gene architecture were classified as the following gene constellations: Overlapping genes were defined as those that overlap in the 5-prime, exonic, or intronic regions. Chromatin co-clustering genes were defined as genes that co-clustered within 20 kb of transcriptional territories. If this scheme is applied the stereotypical gene emerges as a rare occurrence (7.5%), slightly varied schemes yielded between ~1%-50%. Moreover, when following our scheme, paired-overlapping genes and chromatin co-clustering genes accounted for 50.1 and 42.4% of the genes analyzed, respectively. Gene constellation was a correlate of a number of functional and evolutionary properties of genes, but its statistical effect was ~1-2 orders of magnitude lower than the effects of recombination, chromosome linkage and protein function. Analysis of datasets on male reproductive proteins showed these were biased in their representation of gene constellations and evolutionary rate Ka/Ks estimates, but these biases did not overwhelm the biologically meaningful observation of high evolutionary rates of male reproductive genes.

**Conclusion:**

Given the rarity of the solitary stereotypical gene, and the abundance of gene constellations that deviate from it, the presence of gene constellations, while once thought to be exceptional in large Eukaryote genomes, might have broader relevance to the understanding and study of the genome. However, according to our definition, while gene constellations can be significant correlates of functional properties of genes, they generally are weak correlates of the evolution of genes. Thus, the need for their consideration would depend on the context of studies.

## Background

The study of the multiple biological dimensions of genes or groups of genes has developed into an exciting research area [1]. For example, in Drosophila, proteins essential to male reproduction have become a paradigm for the effect of sexual conflict on the rate of nucleotide substitutions [2]. This biological dimension has been evaluated with regard to others, such as X-linkage and recombination rates; both known to be correlated to the molecular evolution of genes [1]. This example illustrates how the evaluation of any such biologically relevant aspect of a gene, or groups of genes, is now routinely evaluated relative to a set of properties commonly suspected to affect or to compromise the study.

The ability to conduct multivariate analysis of numerous gene properties and their genomic locations in the broader context of a study in gene evolution was nicely illustrated by the analysis of the relationship between codon usage bias, gene expression, and recombination in Drosophila and other Eukaryote genomes [3,4]. More recently, a virtually comprehensive set of genomic and functional properties that could affect the evolutionary dynamics of genes was analyzed for multiple Drosophila genomes [1]. These and similar such studies illustrated the difficulty to distinguish mere correlation from causation. For example, the relationships between gene expression levels, codon usage bias, and recombination rates could be a by-product of GC content variation and/or gene density [3,5].

Despite these and similar complications that arise from a wide array of correlations, such analyses illustrated the ability to address questions that could not have been resolved in the pre-genomic era. For instance, it appears that the positive or inverse relationship of recombination rates with the rates of non-synonymous substitutions per non-synonymous site (Ka) can be resolved, in part, once genes are separated into two classes: fast evolving genes that experience positive selection and constrained genes under strong purifying selection [1]. This example illustrates how the grouping of genes by a particular property can help unveil important biological phenomena.

With complete genome sequences in hand, the power to detect properties that unify or separate large groups of genes has greatly increased. However, while some properties affecting gene evolution have long been studied, other properties have yet to receive attention. Post-genomic studies continue to reveal many properties affecting the evolution of genes, including properties that in the past were viewed as too exceptional to be considered of broad relevance to the evolution of genes and genomes. One such property is the coding sequence overlap between two genes. This property has previously been studied in the field of experimental molecular biology in special cases [6] and recently has been more frequently reported in Eukaryote genomes [7,8], but gene constellation has not been noted as a general feature of Eukaryotic genomes.

A bird's-eye view of the genome has now begun to reveal that gene constellations once thought to be exceptional in large Eukaryotic genomes should perhaps be considered of more general importance [7-10]. For instance, one of the first such discoveries published on a larger Eukaryotic genome, *D. melanogaster*, showed that genes that co-cluster within 20-200 kilobases (kb) (median 100 kb) can generate correlations in the pattern and timing of gene expression [10-14]. This indicates that genes that co-cluster in this way should not be viewed as independent, at least in the context of some research questions. Genome biologists have begun to embrace such co-clustering of genes within transcriptional territories as a notable biological feature of the genome, even in large Eukaryotic genomes. Operationally, for the purpose of our study we refer to deviations from the stereotypical gene architecture as gene constellations because these deviations involve the physical overlap of pairs of genes or their spatial proximity within distances that cover transcriptional territories.

**Figure 1:**
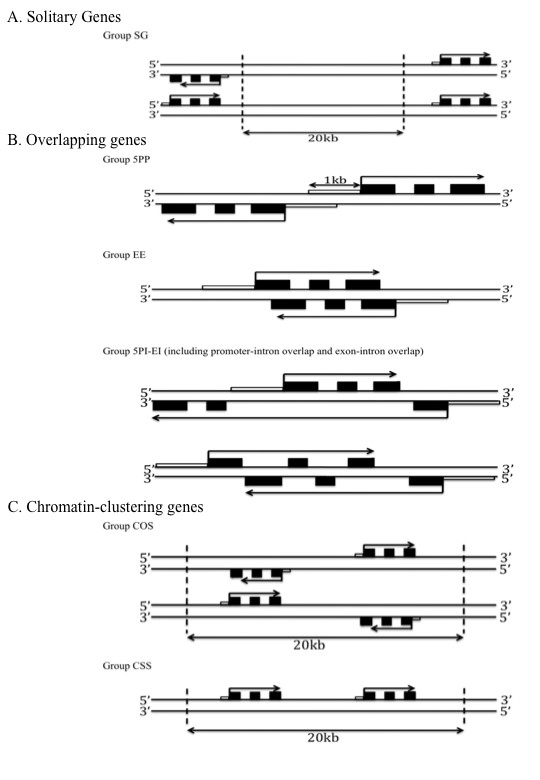
**The gene constellations found in the *Drosophila *genome**. **(A) **Solitary genes were defined as genes without adjoining or overlapping genes on either strand within 20 kb (Group SG). **(B) **Overlapping genes were defined as genes whose 5-prime region is in full or partial overlap with the 5-prime region of another gene (group 5PP), or genes whose coding regions (exons) fully or partially overlap with the coding region (exons) of another gene (group EE), or genes whose coding region (exons) fully overlaps with the introns of another gene (Group 5PI-EI). **(C) **Chromatin co-clustering genes were defined as genes that co-locate within 20 kb of one another on opposite strands (Group COS), or as genes that co-locate within 20 kb of one another on the same strand (Group CSS). This group was considered because it has been shown that co-clustering within such distances results in non-independent expression [11,13].

The goal of our study was to examine if gene constellations should be considered as a genomic property affecting functional and evolutionary properties of genes in the Drosophila genome. Most current analyses of molecular evolution and population genetics in Drosophila still implicitly refer to the stereotypical gene as depicted in textbooks (e.g., [15], Figure 1A). In higher eukaryotes, we typically think of such a gene as a solitary single-copy gene that is, for all practical purposes, independently regulated (i.e. gene regulation is not overwhelmed by regional effects). However, as we show, the stereotypical gene is an exception in the Drosophila genome, not the rule. Thus, we pose the question of whether functional and evolutionary analyses of Drosophila genes could be biased if the genes analyzed deviate from this stereotypical gene architecture. We also want to ask what we can learn about genome biology if gene constellation is considered a genomic property. From the outset of the study, it was clear that both the scheme to classify gene constellations and the list of properties chosen for analyses were not meant to be exhaustive. As indicated above, results of our analyses are interpreted in two contexts. First, gene constellation is discussed in the context of the biology of genes, or groups of genes. Notably, as one would expect based on results obtained from previous genome analyses that have shown that biologically meaningful correlations among multiple properties generally are, albeit significant in statistical terms, generally subtle in magnitude [1,3,4], that any gene constellation effects would emerge as subtle also. Second, gene constellation is discussed as a parameter that might bias hypothesis testing if ignored. In both cases, the effect of gene constellation on the functional and evolutionary properties of genes is discussed relative to that of better-understood properties, including recombination rate, X versus autosomal linkage, and protein function.

## Results

### Enumeration of gene constellations

**Figure 2:**
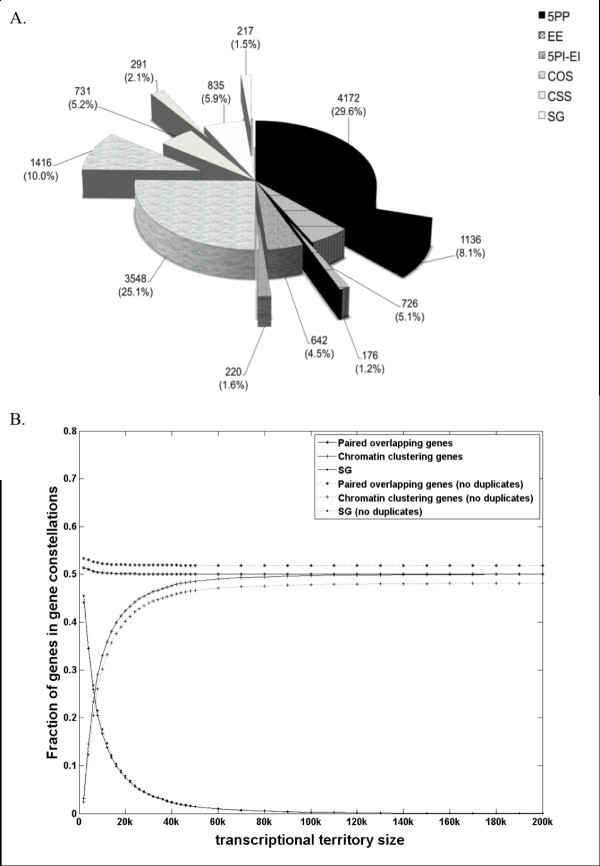
**Enumeration of gene constellations in the *Drosophila *genome**. **(A) **Numbers and percentages of genes that resulted from the application of the scheme depicted in Figure 1. Duplicated genes are shown as pie slices. **(B) **Plot of gene abundance for each constellation versus genomic distances over which co-clustering genes have shown correlations in expression (transcriptional territories; range 20-200 kb, median 100 kb [11,13]). Gene abundances prior to and following removal of gene duplicates are shown as solid lines and dashed lines, respectively.

Deviations from the stereotypical gene architecture were defined as three main groups of gene constellations (c.f. methods and Figure 1). Of 14,110 *D. melanogaster *genes considered, 1,052 (~7.5% of total) met our criteria for stereotypical solitary (SG) genes (Figure 2A). Overlapping genes (5PP, EE, 5PI-EE) accounted for 7,072 of all genes (~50.1%), of which the 5PP gene constellation was most abundant (5,308 genes, ~37.6% of total). The second largest group consisted of 5,986 chromatin co-clustering genes (42.4%; COS and CSS). Within this group, the COS gene constellation was most abundant (4,964 genes, 35.2%). We provide the list of these genes and their classifications in Figure 1 and in Additional File 1, and with the caveats discussed below, we suggest that this annotation list could be used as is or in modified forms to test datasets for compositional biases.

The scheme described above to classify genes is not the only possible classification. For example, we considered transcriptional territories as a biological reality, which may be important to some studies but less so to others. However, while we did consider transcriptional territories, we did not wish to overemphasize their influence, and thus, we used the lowest threshold of 20 kb (in terms of distance separating genes) to account for their presence. To examine the effect of applying this lower threshold we plotted the fraction of genes within each constellation as a function of transcriptional territory sizes up to 200 kb (the estimated maximum distance between genes co-clustering as transcriptional territories [11]). When transcriptional territories (chromatin co-clustering genes) are considered as part of a gene constellation classification scheme, the expansion of transcriptional territories beyond distances >20 kb decreases the percentage of SG estimated (Figure 2B). The number of paired overlapping genes remains unaltered, unless distances < 1 kb are used to classify overlapping and chromatin clustering genes, in which case single genes would account for ~50% of all genes in the *D. melanogaster *genome. Thus, gene constellations that deviate from the stereotypical gene architecture as defined by us in the methods section, rather than the stereotypical solitary gene, remain a widespread feature of the Drosophila genome even when classification schemes ignoring transcriptional territories are utilized.

### Genomic and functional correlates of gene constellations

#### Gene duplication

Whether or not a gene has a duplicated copy in the genome can affect its functional properties and evolutionary dynamics. For example, functional similarity amongst duplicated genes is higher when compared to randomly paired singleton genes [16].

We observed significant (Chi-square test; P-value < 0.001) differences in the number of *D. melanogaster *gene duplications identified in each constellation when compared to the average singleton/duplicate gene ratio of 3.083 calculated for the whole *Drosophila *genome (Figure 2A). Specifically, the singleton/duplicate gene ratios were 3.848 for SG, 3.673 for 5PP, and 4.125 for EE, indicating that duplicates were underrepresented is these groups; the same ratios were 2.919 for 5PI-EI, 2.506 for COS, and 2.512 for CSS, indicating that duplicates were overrepresented for these groups (Table 1).

**Table 1:** Genomic and functional correlates of gene constellations^**a**^.

Group	Recombination rate (X-/Auto)	X/Autosomal linkage	***Sim***_***Rel***_** All**_**BM**_	***Sim***_***Rel***_** BP**_**BM**_	***Sim***_***Rel***_** MF**_**BM**_	***Sim***_***Rel***_** CC**_**BM**_	Expression correlation
**SG**	2.782(± 0.923)/1.451(± 1.365)	155/680	0.165 (± 0.173)	0.165* (± 0.218)	0.101* (± 0.167)	0.141 (± 0.205)	0.027 (± 0.551)
**5PP**	2.755.*(± 0.959)/1.646*(± 1.292)	671/3501	0.326** (± 0.286)	0.289** (± 0.306)	0.182** (± 0.255)	0.258** (± 0.299)	0.278** (± 0.540)
**EE**	2.634(± 1.033)/1.645(± 1.270)	94/632*	0.217** (± 0.184)	0.211** (± 0.239)	0.133** (± 0.210)	0.196** (± 0.231)	0.154** (± 0.596)
**5PI-EI**	2.933*(± 0.859)/1.593(± 1.332)	104/538	0.114** (± 0.180)	0.171 (± 0.247)	0.113 (± 0.198)	0.085** (± 0.163)	0.086 (± 0.525)
**COS**	2.704(± 0.998)/1.676*(± 1.311)	582/2966	0.352** (± 0.323)	0.292** (± 0.330)	0.198** (± 0.286)	0.258** (± 0.329)	0.097** (± 0.578)
**CSS**	2.721(± 0.964)/1.706*(± 1.335)	142/589*	0.304** (± 0.300)	0.276** (± 0.320)	0.195** (± 0.290)	0.223** (± 0.296)	0.119** (± 0.581)

**Genomic average **^**b**^	2.742(± 0.968)/1.643(± 1.308)	1748/8906	0.165 (± 0.173)	0.166 (± 0.218)	0.101 (± 0.167)	0.141 (± 0.205)	0.027 (± 0.551)

^**a**^c.f. Additional File 1 for data and Additional file 4 for a summary of sample sizes.

^**b**^Average values, except for the ratio of X- and autosomal genes, where the value provided should be considered the genome wide ratio.

* and ** indicate significant deviation from the genomic average at P-value ≤ 0.05 and P-value ≤ 0.001, respectively (Chi-square test or two-sided Mann-Whitney test, not corrected for multiple testing).

Therefore, to enhance our ability to study the effect of gene constellation on functional and evolutionary properties while reducing the confounding effect of gene duplication on these properties, duplicated genes (N=3,456) were excluded from further analyses. The relative abundances (in fraction) of genes in each constellation before and after the removal of gene duplicates from the data remain similar (Figure 2B).

#### Chromosomal location

The location of a gene on the X chromosome versus the autosomes may have an effect on its functional properties and evolutionary dynamics [17,18]. For example, recessive mutations are exposed to selection in the hemizygous X of males.

Gene constellations occur with expected frequency on the X and on the autosomes of *D. melanogaster *as predicted by their overall frequency (Chi-square test; P-values ≥ 0.05; Table 1), except for a small deficiency or excess on the X of the EE (~5%; P-value = 0.012) and CSS (~5%; P-value = 0.028) constellations, respectively. X versus autosomal linkage was considered during all analyses.

#### Recombination rates

The recombination rate is a correlate of the effect of genetic drift and selection on levels of genetic variation within species and the rates of molecular evolution between species [19,20].

In *D. melanogaster*, the average recombination rates of the X-chromosome exceed the recombination rates of the autosomes [18] (Table 1). Gene constellations 5PP, COS and CSS were found more frequently (P-value < 0.05) in regions of higher average recombination rates on the autosomes (Table 1). Genes on the X belonging to groups 5PP and 5PI-EI were found mostly in regions showing higher recombination rates (P-value < 0.05). Recombination rate variation was considered in the examination of the evolutionary properties of gene constellations (see below).

#### Functional similarity

The function of proteins can be correlated to the type and strength of the selection on their underlying genes [19]. For example, it is generally believed that genes involved in the early development of the fly are under purifying selection [21], whereas genes involved in male reproduction diversify rapidly between species [2].

We investigated whether pairs of genes in each *D. melanogaster *constellation display similarities in their function, based on their associated Gene Ontology (GO) terms (c.f. Additional File 2 for GO annotations). This analysis indicates that genes occurring in particular constellations are subject to similar selection pressures. We present results based on the analysis of functional similarity calculated from the Relevance Semantic Measure (*Sim*_*Rel*_) and the *GO*_*BM *_measure (including *BP*_*BM*_, *MF*_*BM*_, *CC*_*BM *_and *All*_*BM *_estimated across GO roots) [22,23]. We found that genes belonging to the groups SG and 5PI-EI were representative of the genomic average (randomly paired genes) in terms of pairwise functional similarity (Table 1). Overlapping genes (5PP and EE) and chromatin co-clustering genes (COS and CSS) showed higher than expected functional similarities. Results based on the analysis of other functional similarity measures (c.f. methods) led to the same conclusion (data not shown). This bias in gene function was considered when examining the evolutionary properties of gene constellations (see below).

#### Functional enrichment

**Figure 3:**
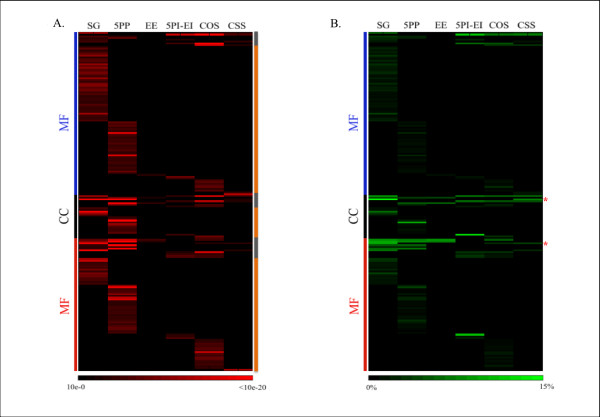
**Heat maps depicting the enrichment of GO terms in each gene constellation**. **(A) **The significance of GO term overrepresentations in each gene constellation. The scale bar at the bottom of the graph depicts the range of significance values. GO terms uniquely overrepresented in gene constellations are marked in the orange right side bar, and GO terms shared by two or more gene constellations are marked in gray. **(B) **The enumeration of genes with associated overrepresented GO terms. The scale bar at the bottom of graph depicts the percentages of genes belonging to each gene constellation that are associated with the GO term. The * depict two examples where gene constellations varied greatly in terms of the abundance (percentage) of genes associated with this GO term (GO:0005634 Nucleus (CC): SG (14.73%), 5PP (0.51%), CSS (7.63%); GO:0003700 transcription factor activity (MF): SG (10.17%), COS (2.7%), CSS (3.91%). C.f. Additional file 2 for data.

Enrichment of GO terms could also result in a bias of the evolutionary properties of gene constellations [24]. Analysis of the *D. melanogaster *genome showed that a large number of GO terms were over-represented in each gene constellation (Figure 3A; c.f. Additional file 2 for GO annotations). Many of these were either uniquely overrepresented or enriched in one gene constellation (Figure 3B), indicating that gene constellations could be biased also in terms of gene function.

The overrepresentation of particular GO terms in a gene constellation may bias its evolutionary properties (e.g., Ka/Ks). To illustrate this effect, we calculated Ka/Ks between *D. melanogaster *and *D. pseudoobscura *for all genes in constellations with overrepresented GO terms and contrasted these to the average Ka/Ks computed for gene constellations in general. We observed that the average Ka/Ks (± 1SD) for solitary autosomal genes (SG) with overrepresented GO terms was 0.069 ± 0.07, a lower Ka/Ks when compared to the Ka/Ks of 0.092 when all solitary genes were considered (Mann-Whitney test; P-value = 0.031). Thus, functional bias in the group SG should decrease Ka/Ks.

Similarly, the average Ka/Ks of genes with associated GO terms that were overrepresented in groups 5PP and COS was below the Ka/Ks calculated when all genes were considered (5PP: Ka/Ks = 0.057 ± 0.058, P-value = 1.0 × 10^-6^; COS: Ka/Ks = 0.081 ± 0.079, P-value = 0.005) (c.f. Table 2 for all Ka/Ks values). The gene constellations EE, 5PI-EI, and CSS showed no such bias (all P-values > 0.05). Therefore, enrichment in function was taken into consideration when examining evolutionary properties (see below).

**Table 2:** Evolutionary correlates of gene constellations^a^.

			African population			American population					
						
Group	CAI	Ka/Ks	**θ**_**W**_	**θ**_**π**_	Tajima's D	**θ**_**W**_	**θ**_**π**_	Tajima's D	**F**_**ST**_	**Expr-Div**^**c**^	**Q**_**ST**_
**Genes on the autosomes**											

**SG**	0.233** (± 0.039)	0.092 (± 0.109)	0.008 (± 0.002)	0.006 (± 0.001)	-1.023 (± 0.363)	0.003 (± 0.001)	0.004 (± 0.001)	0.199 (± 0.689)	0.125 (± 0.097)	2.7%	0.427 (± 0.296)
**5PP**	0.252** (± 0.039)	0.073* (± 0.079)	0.007 (± 0.003)	0.005 (± 0.002)	-1.058 (± 0.488)	0.003 (± 0.001)	0.003 (± 0.002)	0.514 (± 0.807)	0.153 (± 0.103)	3.5%	0.362 (± 0.272)
**EE**	0.250* (± 0.037)	0.075* (± 0.100)	0.007 (± 0.002)	0.006 (± 0.002)	-0.928 (± 0.358)	0.003 (± 0.002)	0.003 (± 0.002)	0.380 (± 0.519)	0.142 (± 0.151)	2.5%	0.386 (± 0.275)
**5PI-EI**	0.236** (± 0.040)	0.097 (± 0.108)	0.007 (± 0.002)	0.005 (± 0.002)	-1.010 (± 0.664)	0.003 (± 0.001)	0.003 (± 0.001)	0.529 (± 0.663)	0.121 (± 0.099)	6.8%*	0.401 (± 0.271)
**COS**	0.241* (± 0.043)	0.098** (± 0.103)	0.008 (± 0.003)	0.006 (± 0.002)	-0.991 (± 0.528)	0.003 (± 0.002)	0.004 (± 0.002)	0.557 (± 0.828)	0.125 (± 0.109)	4.7%	0.373 (± 0.283)
**CSS**	0.236** (± 0.041)	0.107** (± 0.117)	0.009 (± 0.003)	0.007 (± 0.003)	-1.038 (± 0.555)	0.004 (± 0.003)	0.004 (± 0.003)	0.098 (± 0.670)	0.111 (± 0.160)	4.2%	0.411 (± 0.294)

**Genomic average**^**b**^	0.245 (± 0.041)	0.085 (± 0.095)	0.008 (± 0.003)	0.006 (± 0.002)	-1.020 (± 0.510)	0.003 (± 0.002)	0.003 (± 0.002)	0.469 (± 0.781)	0.134 (± 0.110)	4.0%	0.377 (± 0.279)

**Genes on the X chromosome**											

**SG**	0.239* (± 0.051)	0.103 (± 0.120)	0.009 (± 0.003)	0.007 (± 0.002)	-0.980 (± 0.455)	0.002 (± 0.001)	0.002 (± 0.001)	0.105 (± 1.083)	0.226 (± 0.125)	0.6%	0.412 (± 0.318)
**5PP**	0.263** (± 0.040)	0.074* (± 0.074)	0.007 (± 0.003)	0.006 (± 0.002)	-0.891 (± 0.486)	0.002 (± 0.001)	0.003 (± 0.001)	0.433 (± 0.857)	0.233 (± 0.123)	1.9%	0.379 (± 0.295)
**EE**	0.255 (± 0.037)	0.080 (± 0.056)	0.008 (± 0.001)	0.007 (± 0.001)	-0.886 (± 0.279)	0.003 (± 0.001)	0.004 (± 0.001)	0.625 (± 0.639)	0.219 (± 0.061)	1.1%	0.416 (± 0.309)
**5PI-EI**	0.247 (± 0.038)	0.090 (± 0.081)	0.009 (± 0.002)	0.007 (± 0.002)	-0.996 (± 0.363)	0.003* (± 0.001)	0.004 (± 0.000)	0.509 (± 0.839)	0.209 (± 0.086)	1.0%	0.364 (± 0.276)
**COS**	0.254 (± 0.045)	0.096 (± 0.098)	0.008 (± 0.003)	0.007 (± 0.003)	0.814 (± 0.547)	0.002 (± 0.002)	0.003 (± 0.002)	0.453 (± 0.851)	0.215 (± 0.185)	2.6%	0.375 (± 0.268)
**CSS**	0.242* (± 0.037)	0.096 (± 0.112)	0.008 (± 0.003)	0.006 (± 0.003)	-0.868 (± 0.453)	0.003 (± 0.001)	0.003 (± 0.001)	0.339 (± 0.698)	0.189 (± 0.107)	2.8%	0.344 (± 0.285)

**Genomic average**^**b**^	0.255 (0.043)	0.085 (± 0.088)	0.008 (± 0.003)	0.006 (± 0.002)	-0.877 (± 0.489)	0.002 (± 0.001)	0.003 (± 0.002)	0.400 (± 0.857)	0.220 (± 0.141)	2.0%	0.378 (± 0.286)

^**a **^c.f. Additional File 1 for data and Additional file 4 for a summary of sample sizes.

^**b**^Average values except for the measure for expression divergence (Expr-Div), where the value provided should be considered the genome wide proportion of differentially expressed genes.

^**c **^Expression Divergence: the percentage of differentially expressed genes between *D. melanogaster *and *D. yakuba*.

* and ** indicate significant deviation from the genomic average at P-value ≤ 0.05 and P-value ≤ 0.001, respectively (Chi-square test or two-sided Mann-Whitney test, not corrected for multiple testing).

#### Correlation of gene expression

The intensity and tissue specificity of gene expression can be correlated to the evolutionary properties of genes [10]. For example, selection for translational efficiency is one possible explanation for the connection between gene expression and codon usage bias [25]. Male-biased expression of genes is correlated to the rate of rapid non-synonymous site substitution of genes [10,13,26].

Analysis of *D. melanogaster *gene expression showed that pairs of solitary genes (SG) and overlapping genes (5PI-EI) were representative of the genomic average (Table 1). In contrast, overlapping genes of type 5PP and EE, and chromatin co-clustering genes (COS and CSS) showed higher than expected pairwise correlations of gene expression (Table 1).

### Evolutionary correlates of gene constellations

#### Conservation of gene constellation

Persistence and turnover of gene constellations may be indicative of evolutionary processes and/or technical issues related to gene annotation. For example, ~4500 *D. melanogaster *genes (including duplicated genes) in different constellations have no consensus ortholog in *D. pseudoobscura*. This might indicate rapid turnover of genes in particular gene constellations and/or, that these genes are more difficult to annotate and assign as orthologs than others. We compared conservation of gene constellations between *D. melanogaster *and *D. pseudoobscura*. The latter species was chosen because one-to-one assignment of orthologs to *D. melanogaster *genes is well established and this information is accessible (Inparanoid; [27]).

**Figure 4:**
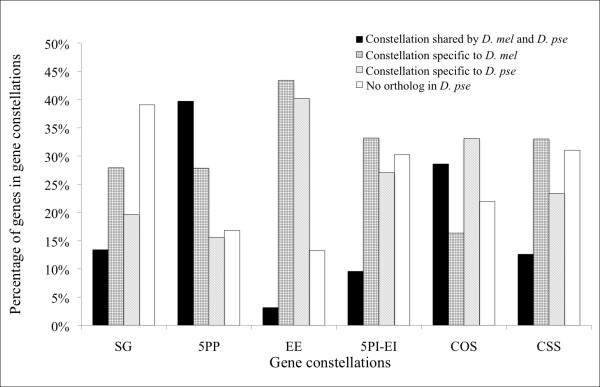
**Enumeration of orthologous genes in each constellation that are shared or are unique to *D. melanogaster *(*D. mel*) and *D. pseuoobscura *(*D. pse*)**. Note that it is uncertain whether conservation of gene constellation is a result of evolutionary processes and/or technical factors (e.g. annotation of orthologs; c.f. main text).

We observed two patterns amongst the 7546 orthologous gene pairs without *D. melanogaster *duplicate genes (Figure 4, c.f. Additional File 3 for results when duplicated genes were included in the analysis). First, for most groups, only a low fraction of genes are conserved and/or annotated in the same constellation in both species, most genes are found and/or annotated in a species-specific constellation (SG, EE, 5PI-EI, CSS). In contrast, the two most abundant gene constellations 5PP and COS contain similar or higher fractions of genes that are conserved between species than genes in species-specific constellations.

We found 3126 *D. melanogaster *genes that had no orthologs in *D. pseudoobscura*. These genes may not be annotated, or assigned, or may not be present or recognizable in *D. pseudoobscura *due to gene loss/fusion or other evolutionary phenomena such as evolutionary rate acceleration resulting from positive selection (duplicated genes excluded) (c.f. also [28]). The percentages of genes with missing orthologs in each constellation were as follows: SG (13.0%), 5PP (26.6%), EE (5.2%), 5PI-EI (8.5%), COS (37.2%) and CSS (9.5%). The fact that genes that should be relatively straightforward to annotate (e.g., SG, COS, CSS) in *D. melanogaster *are overrepresented among those missing orthologs in *D. pseudoobscura *might indicate that these genes now are in certain gene constellation that renders annotation difficult. The fact that the missing orthologs cannot be studied at present makes it difficult to identify the causes of their delayed or failed annotation. However, our results do indicate that the evolution of genes can involve changes in gene constellation. The evolutionary and technical factors explaining these merit detailed analyses that are outside the scope of this manuscript.

#### Codon usage bias

Codon usage bias is used as a proxy measurement for the efficacy of weak selection [3,4].

The average codon usage bias (as measured using the codon adaptation index; CAI) of the most abundant gene constellation (5PP) exceeds the average CAI of the *D. melanogaster *genome (Table 2). The second most abundant gene constellation (COS) has a codon usage bias below the average. The remaining gene constellations also showed higher (EE) or lower (SG, 5PI-EI, CSS) bias when compared to the genome average.

Thus, the two most abundant gene constellations (5PP and COS) potentially bias inferences of codon usage in an upward and downward direction, respectively, depending on which of the two might be overrepresented in a given dataset. We would expect that these overlapping gene constellations are more abundant in genomic regions of high gene density [3]. Thus, the higher than average codon usage bias observed for the gene constellations 5PP and EE is consistent with results of an earlier study that reported high codon usage bias in regions of high gene density [3].

#### DNA sequence divergence

We investigated whether the gene evolution between *D. melanogaster *and *D. pseudoobscura *could be affected by the grouping of genes in different constellations. For example, nucleotide sites in genes whose exons overlap might be under dual selective constraint (measured as the ratio of non-synonymous to synonymous nucleotide site substitution rate between species, Ka/Ks), when compared to solitary genes, where nucleotides encode information for one gene only.

Gene constellations with functional overlap (5PP or EE) displayed reduced Ka/Ks relative to the genome average (Table 2). In contrast, overlap between functional and presumably non-functional regions (5PI-EI) was not associated with reduced Ka/Ks. Elevated Ka/Ks measurements were observed for chromatin clustering genes (COS, CSS). The Ka/Ks ratios of solitary genes (SG) were average. Above trends were unsupported when the X chromosome was analyzed, though we did note the low Ka/Ks of genes in the 5PP group on the X (Table 2).

Our results are consistent with the idea of gene constellations representing a biological feature of genes that can affect (or is correlated with) nucleotide substitution rates of genes.

#### DNA sequence polymorphism

Genetic polymorphism measures are useful for inferring the influences of selection, drift, and demographics on gene frequencies in populations [20]. Such studies are involved because they need to consider (assume) complex demographic models and an array of other variables (e.g., recombination rates). The subtle effects caused by each variable often leads to competing interpretations of the results.

We examined whether gene constellation groupings are correlated with population genetic measures at the level of genetic diversity (measured as θ_W_, θ_π_, and a composite measure thereof Tajima's D) when *D. melanogaster *populations are analyzed (Table 2). F_ST _between African and North American populations of *D. melanogaster *was estimated to investigate whether gene constellations are correlates of genetic differentiation (measured as Table 2). For example, it is conceivable that population differentiation at exon or promoter overlapping genes differs from other gene constellations in that polymorphisms may affect two genes at a time.

However, we observed that all but one cell in Table 2 were in broad accordance with the genomic average. A limitation of these analyses is the small number of genes studied (c.f. Additional file 4); further, these genes were not chosen at random with respect to recombination rates when they were collected. This will impose limits on the power of multivariate analyses incorporating gene constellation groupings to reveal how this property compares to other, also subtle, properties that influence polymorphism data, e.g., recombination rates (see below).

#### Divergence of gene expression

We investigated whether genes in different constellations displayed different expression levels between species of Drosophila. For example, it is possible that high levels of correlation in expression between overlapping genes, as seen for example in groups 5PP and EE in *D. melanogaster*, may also be manifest as the correlated differential expression between species.

The percentage of genes that are differentially expressed between *D. melanogaster *and *D. yakuba *is higher on the autosomes than on the X chromosome (Table 2). We observed a statistically significant amount of autosomal genes differentially expressed between species for the group 5PI-EI.

#### Variation of gene expression

We investigated whether the differential expression of genes between *D. melanogaster *populations from Africa and Europe varied between gene constellations. The relationship of genes that overlap in functional regions (5PP, EE, e.g.,) or co-cluster in transcriptional territories could result in their constrained evolution between populations. However, none of the gene constellations emerged as significant outliers when we employed Q_ST _as a measure for differentiation of a quantitative character (here: gene expression) between populations (Table 2).

### Quantification of the influence of gene constellation on evolutionary properties

**Figure 5:**
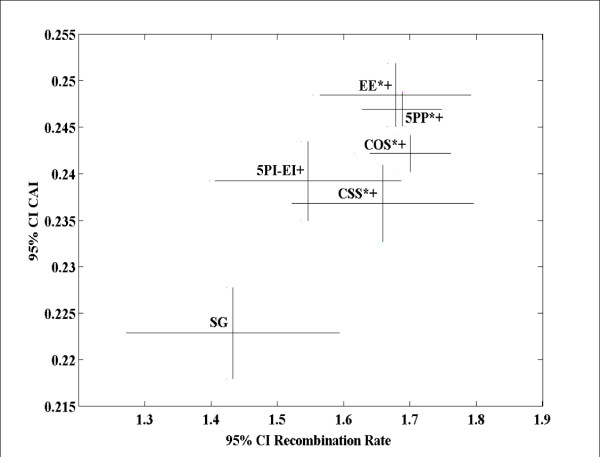
**Codon usage bias (Codon Adaptation Index, CAI) (Y-axis) and recombination rates (X-axis) of autosomal genes following removal of genes associated with overrepresented GO terms**. In all graphs bars represent 95% confidence intervals. Compared to solitary genes, the * indicates a significant difference in recombination rate (X-axis) (P-value ≤ 0.05; Mann-Whitney U tests, two-sided; not corrected for multiple comparisons), and the + indicates a significant difference of the evolutionary property studied (Y-axis) (P-value ≤ 0.05; Mann-Whitney U tests, two-sided; not corrected for multiple comparisons). See Additional File 5 for these analyses prior to the exclusion of genes with over-represented GO terms, and the analysis of X linked genes.

**Figure 6:**
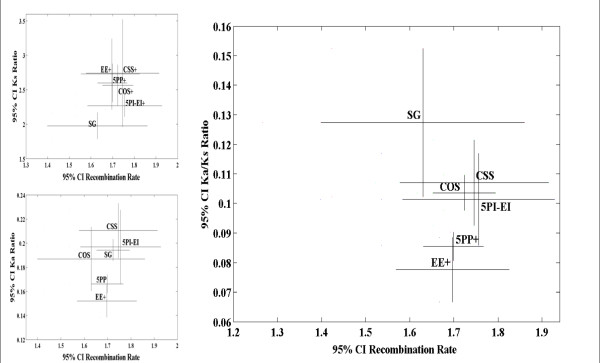
**The ratio of fixation of amino-acid replacement mutations (Ka) over the rate of synonymous mutations (Ks) between *D. melanogaster *and *D. pseudoobscura *(Y-axis) and recombination rates (X-axis) of autosomal genes following removal of genes associated with overrepresented GO terms**. In all graphs bars represent 95% confidence intervals. Compared to solitary genes, the * indicates a significant difference in recombination rate (X-axis) (P-value ≤ 0.05; Mann-Whitney U tests, two-sided; not corrected for multiple comparisons), and the + indicates a significant difference of the evolutionary property studied (Y-axis) (P-value ≤ 0.05; Mann-Whitney U tests, two-sided; not corrected for multiple comparisons). See Additional File 5 for these analyses prior to the exclusion of genes with over-represented GO terms, and the analysis of X linked genes.

**Figure 7:**
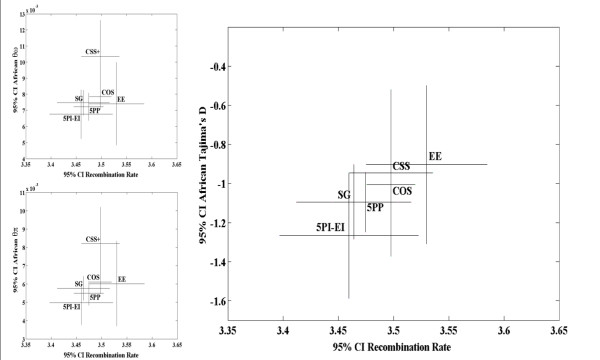
**Genetic variation (Y-axis) and recombination rates (X-axis) of autosomal genes following removal of genes associated with overrepresented GO terms**. Genetic variation estimators (θ_ω_, θ_π_) and their skew (Tajima's D) for the African population of *D. melanogaster*. In all graphs bars represent 95% confidence intervals. Compared to solitary genes, the * indicates a significant difference in recombination rate (X-axis) (P-value ≤ 0.05; Mann-Whitney U tests, two-sided; not corrected for multiple comparisons), and the + indicates a significant difference of the evolutionary property studied (Y-axis) (P-value ≤ 0.05; Mann-Whitney U tests, two-sided; not corrected for multiple comparisons). See Additional File 5 for these analyses prior to the exclusion of genes with over-represented GO terms, and the analysis of X linked genes.

**Figure 8:**
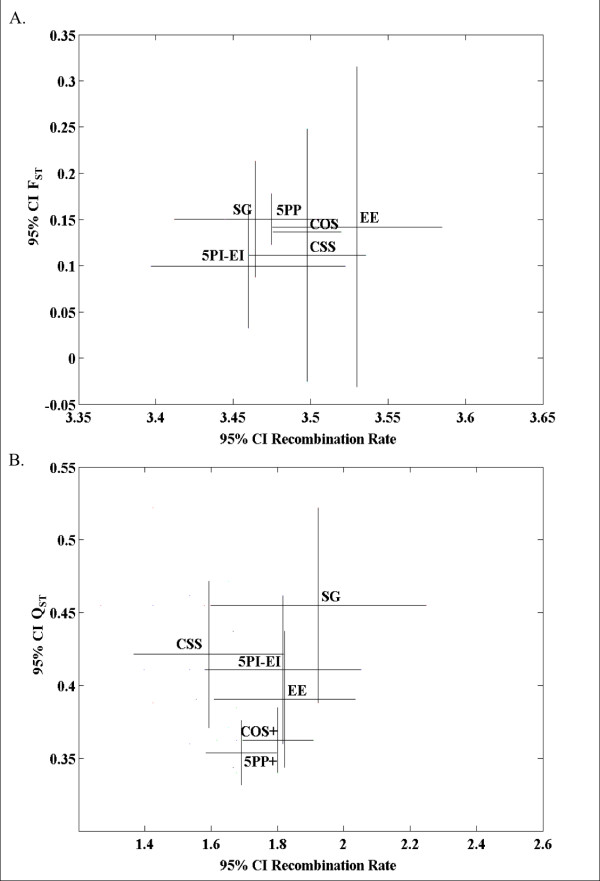
**Population differentiation (Y-axis) and recombination rates (X-axis) of autosomal genes following removal of genes associated with overrepresented GO terms**. **(A) **DNA sequence differentiation (F_ST_) between African and Non-African *D. melanogaster *populations. **(B) **Gene expression differentiation (Q_ST_) between African and Non-African *D. melanogaster *populations. In all graphs bars represent 95% confidence intervals. Compared to solitary genes, the * indicates a significant difference in recombination rate (X-axis) (P-value ≤ 0.05; Mann-Whitney U tests, two-sided; not corrected for multiple comparisons), and the + indicates a significant difference of the evolutionary property studied (Y-axis) (P-value ≤ 0.05; Mann-Whitney U tests, two-sided; not corrected for multiple comparisons). See Additional File 5 for these analyses prior to the exclusion of genes with over-represented GO terms, and the analysis of X linked genes.

We have examined a non-exhaustive set of genomic, functional, and evolutionary properties for correlation with gene constellations as defined by our classification scheme. To infer the relative importance of gene constellation grouping relative to that of other properties, we contrasted the statistical effect of this genomic property on the evolutionary dynamics of genes with other factors with known effects. As points of reference, we considered X- versus autosomal linkage, recombination rate and protein function [1] (Figures 5, 6, 7 and 8) [29,30]. For clarity, in Figures 5, 6, 7 and 8, we only depict results for autosomal genes (c.f. Additional file 5A for X-linked genes); furthermore, we eliminated genes with overrepresented GO terms to minimize the influence of functional bias (c.f. Additional file 5B for all autosomal genes). Sample sizes available for the study of codon usage bias, Ka/Ks, and gene expression were large and covered nearly the entire spectrum of recombination rates (c.f. Additional file 4). The exception to that was that the subset of genes belonging to the SG group had a higher average recombination rate when compared to all SG (c.f. Figures 5 and 6 and Table 1). Sample sizes available to study polymorphisms data were limited, and genes were sampled from regions of high recombination rates (c.f. Figures 7 and 8), thereby restricting our ability to compare the effect of gene constellation with the effect of recombination rates.

Examination of codon usage bias (CAI) revealed the expected relationship with recombination rate (Figure 5), and thus, was not indicative of a strong effect of gene constellation on CAI. 5PI-EI genes were the exception in that their recombination rates were not significantly higher than those of solitary genes, but the CAI was increased. However, during multivariate analysis, in addition to X- versus autosomal linkage, gene function, and recombination rates, a significant effect of gene constellation on CAI was detected (Table 3). However, the impact of gene constellation on CAI was two to one order of magnitudes lower (measured as R) when compared to the effect of gene function and recombination rates, respectively (Table 3).

**Table 3:** Multivariate model fitting of genomic- and functional properties^a ^of genes to their evolutionary properties^b^.

Evolutionary property (sample size)	Genomic and functional properties	Fitted model	R	P-value
**Codon usage bias (CAI) **(N = 10118)	X- versus autosomal linkage (Chr.)	-0.027 + 0.256 Chr.	0.096	1.0 × 10^-6^
	Gene Ontology (GO)	-0.077 + 0.256 Chr. + 0.123 GO	0.114	1.0 × 10^-6^
	Recombination rate (Rec.) and gene constellation (Const.)	0.051 + 0.222 Chr. + 0.114 GO + 0.041 Rec. - 0.008 Const.	0.129	1.0 × 10^-6^

**Ka/Ks **(N = 7425)	Gene Ontology (GO)	0.103 - 0.246 GO	0.121	1.0 × 10^-6^
	Gene constellation (Const.)	-0.115 - 0.222 GO + 0.014 Const.	0.144	1.0 × 10^-6^

**Tajima's D **(N = 461)	Recombination rate (Rec.)	0.239 - 0.301 Rec.	0.160	6.0 × 10^-4^
	Gene Ontology (GO)	0.154 - 0.293 Rec. + 0.184 GO	0.184	4.0 × 10^-4^

**F**_**ST **_(N = 461)	X- versus autosomal linkage (Chr.)	-0.286 + 0.657 Chr.	0.326	1.0 × 10^-6^

**Q**_**ST **_(N = 3238)	No fitting model			

^a^Subset of properties chosen from Table 1 whose effects on the evolutionary dynamics of genes commonly appreciated

^b^C.f. Table 2

^c^Ka and Ks analyzed separately (c.f. main text for results)

^d^θ_W _and θ_π _analyzed separately (c.f. main text for results)

Contrary to expectations Ka/Ks did not increase with increasing recombination rates (Figure 6). During multivariate analysis, protein function and gene constellation groupings emerged as significant model properties (Table 3). The relative effect of gene constellation on Ka/Ks was weak (~7%) of the effect that gene function has on Ka/Ks (Table 3).

We were unable to identify a fitting model for Ks, even though higher recombination rates appeared to be associated with higher Ks (Figure 6 left upper panel). The analysis of Ka indicated that exon-overlap and 5-prime overlap are correlates of low non-synonymous nucleotide site evolution (Figure 6 lower panel). Multivariate analysis on Ka supported a model that involved X- versus autosomal linkage, gene function, and gene constellation (fitting model: -0.207 + 0.018 Const. - 0.182 protein function + 0.156 Chr.; R = 0.153, P-value = 1.0 × 10^-6^).

None of the properties studied had an effect when we analyzed θ_ω _and θ_π _as genetic polymorphism estimators of the African population, the population thought to be closest to mutation-drift equilibrium [31]. For the American population, X- versus autosomal linkage emerged as a property significantly affecting θ_ω _(fitting model: 0.163 - 0.374 Chr.; R = 0.186, P-value = 6.0 × 10^-5^) and θ_π _(Figure 7). Multivariate analysis identified gene constellations as a model feature affecting θ_π _in the American population of *D. melanogaster *(fitting model: -0.102 - 0.393 Chr. + 0.019 Const.; R = 0.228, P-value = 5.0 × 10^-6^). Analysis of Tajima's D identified recombination rates and gene function as significant properties in the African *D. melanogaster *population (Table 3). Finally, no relationship between F_ST _and gene constellation was supported, but X- versus autosomal linkage emerged as a significant property (Figure 8A; Table 3). Thus, in terms of its effect on population genetic parameters, gene constellation generally had no effect. The fact that genes were collected originally for genomic regions representing a rather narrow range of high recombination rates explains why recombination generally did not emerge as an important variable. Finally, the complexity of interpreting results at the level of population genetic parameters, as well as the low sample size, may have hampered these analyses.

Finally, increasing recombination rates were associated with lower levels of gene expression differentiation between *Drosophila *populations (Figure 8B), but multivariate analysis did not support any significant model.

### Relevance of observations to hypothesis testing

We showed that gene constellation groupings are correlated to a number of genomic, functional and evolutionary properties (Tables 1-2). The effect during multivariate analysis was about 1-2 orders of magnitude weaker than genomic and functional properties (Table 3). Our observations are relevant to datasets that are biased in their representation of various gene constellations, as they may also be biased in their evolutionary properties, therefore potentially leading to type I or type II errors.

As an example, male reproductive genes in Drosophila tend to evolve at higher Ka/Ks than the genomic average, and datasets used to test this hypothesis tend to consist of small to medium sized collections of genes. We chose two representative studies, chosen owing to their high quality of data and their comprehensive analyses. We tested for compositional bias in terms of gene constellations sampled, and calculated how this could bias the biological hypothesis tested (here: male sexual reproductive role and elevated Ka/Ks of genes).

The relative composition in terms of gene constellations amongst 748 genes with male biased gene expression patterns [32] was uneven (Chi-square test; P-value = 5.8 × 10^-8^). We found a deficiency of genes belonging to group 5PP and an excess of genes belonging to group COS (Table 4A). Similarly, a dataset of 50 Acp (male accessory gland protein) genes [33] was characterized by a deficiency of 5PP genes and an excess of COS genes (Table 4B). These compositional biases with regard to gene constellations could have biased the Ka/Ks upwardly, since the overrepresented gene constellation (COS) and the underrepresented gene constellation (5PP) tend to have higher and lower Ka/Ks, respectively (Table 2).

**Table 4:** Testing for biased representation of gene constellations in empirical datasets of male reproductive genes.

Gene constellations						
SG	5PP	EE	5PI-EI	COS	CSS	Total
A. Genes with male biased expression*						

5 (2.6)	59 (91.9)	16 (15.9)	6 (3.9)	67 (41.8)	8 (5.0)	161 (161)

B. Acp genes*						

2 (1052)	9 (5308)	1 (902)^‡^	3 (862)	29 (4964)^‡^	6 (1022)	50 (14110)

A. Genes with male biased expression [32] and B. Acp (male reproductive accessory gland) genes [33]. In A * indicates the significant deviation (two-sided Chi-square test, P-value ≤ 0.01) of the observed number of genes from the expected number of genes in each gene constellation. In **B **^‡ ^indicates the significant over-/under- representation of genes (two-sided two sample proportional test, P-value ≤ 0.05) with respect to the numbers of genes found in the genome.

Multivariate analysis applied to the dataset of 50 Acp genes [33] showed that gene constellation was a significant yet low-effect model feature for Ka/Ks, in addition to the function of genes as Acp proteins (Table 5). For the analysis of Ka, X- versus autosomal linkage was supported as an additional model feature. However, no model could be fitted to the Ks data. Using the published Ka/Ks values [33] we observed that the average Ka/Ks (0.279) of the underrepresented 5PP genes was lower than the average Ka/Ks (0.371) of the overrepresented COS genes, suggesting the Ka/Ks in the whole dataset could be biased in an upward direction due to the compositional bias of gene constellations sampled. As the authors suggested that the molecular evolutionary rates of Acp genes evolve at rates faster than the genomic average; this bias could result in a type I error in these studies.

**Table 5:** Multivariate model fitting of genomic- and functional properties^a ^of genes to their evolutionary properties^b ^for male accessory gland protein genes [33].

Evolutionary property	Genomic and functional properties	Fitted model	R	P-value
**Ka/Ks**	Gene constellation (Const.)	-0.251 + 0.017 Const.	0.095	1.0 × 10^-6^
	Gene function as male accessory gland protein (Acp)	-0.250 + 1.309 Acp + 0.017 Const.	0.103	1.0 × 10^-6^

**Ka**	Gene constellation (Const.)	-0.294 + 0.020 Const.	0.111	1.0 × 10^-6^
	X- versus autosomal linkage (Chr.)	-0.318 + 0.157 Chr. + 0.020 Const.	0.125	1.0 × 10^-6^
	Gene function as male accessory gland protein (Acp)	-0.318 + 0.831 Acp + 0.158 Chr. + 0.020 Const.	0.127	1.0 × 10^-6^

**Ks**	No significant model fit			

^a^Subset of properties chosen from Table 1 whose effect on the evolutionary dynamics of genes commonly appreciated

^b^C.f. Table 2

## Discussion

Post-genomic studies now consider the multiple biological dimensions of genes or groups of genes [10,24]. The results of such studies are becoming increasingly informative, but the task of conducting the research is also becoming increasingly complex. Clearly, numerous genomic and functional properties of genes are direct determinants or mere correlates of their evolutionary dynamics, and properties may auto-correlate with one another, as shown comprehensively by, e.g. [1,3,4]. This insight that the evolution of genes is affected by an array of potentially interacting (or correlated) properties suggests that any study focusing on a particular biological dimension needs to also consider an array of other factors.

In essence, genome biologists face the challenge of determining the relative influences of numerous biological facets of a gene in order to understand its most important features. Simply identifying new previously overlooked features that merit investigation should be considered an achievement of the post genome era; even if these features emerge as weak effectors, they may provide insight into the biology of individual genes, groups of genes, or the genome as a whole. Moreover, quantification of the effects of previously overlooked features is important to gauge the bias these may introduce to studies if ignored.

The list of biological properties that should be considered during studies is not standardized, even though it has long been good practice to consider, for example, X- versus autosomal linkage and recombination rates. Other properties also commonly considered include gene duplication and sex-specific expression. The recently published compilation of properties affecting the evolution of genes in *Drosophila *provides guidance to such multidimensional studies [1]. However, the absence of a discussion on gene constellations prompted us to examine if this property deserves consideration also, and if the study of gene constellations would add to the understanding of the complex biology of genes, or adversely, if studies that ignore this property would show bias.

### Relative abundances of gene constellations

Our classification scheme, which considered transcriptional territories but minimized their spatial extent, suggests that the stereotypical single gene architecture should be considered as the exception, rather than the rule, in the *Drosophila melanogaster *genome. Our reference to the rarity of the stereotypical gene is conservative because if higher distances were applied to account for transcriptional territories, their number would be reduced further (Figure 2B). However, as stated, our reference to the rarity of the solitary gene depends on the acceptance of transcriptional territories as a biological reality [11]. Thus, our statement needs to be interpreted in light of their relevance to any particular study. If transcriptional territories were ignored as a genomic feature, or deemed irrelevant in a particular study context, the number of solitary genes would be as high as ~50% (Figure 2B).

Our discovery of the rarity of the stereotypical solitary gene (Tables 1 and 2) might be of relevance to the design of molecular evolution and population genetic studies and to the possibility that previous studies ignoring this genomic property suffered from bias. Conceivably, researchers would pick solitary genes for analysis, because intuitively, overlapping genes would seem a poor choice. Chromatin-clustering genes would also be less favored in an attempt to avoid the effect of correlated expression patterns of co-clustered genes [12,13] or to space genes along the chromosome to avoid correlations in recombination rates [16]. Such a study design would likely end up with a collection of stereotypical solitary genes, which, according to our results, can differ from other genes e.g. in terms of codon usage bias and Ka/Ks. Thus, such a collection of stereotypical genes may not be representative of the overall genome.

We feel that the classification and resulting enumeration of 5PP, EE, and 5PI-EI genes are unlikely to be contentious, except that the distance between promoter/enhancer overlapping genes may be varied (reduced to <1 kb). Chromatin clustering genes COS and CSS are defined from previous results describing transcriptional territories, but this is also subject to other uncertainties. These uncertainties refer to the relevance of such transcriptional territories to any particular study context. Moreover, the number of genes that co-cluster in transcriptional territories varies as a function of the distances applied to classify them.

A second concern regards the distinction of 5PP and EE genes. The 5-prime region of a gene may overlap with the 5-prime region of another gene located in its 5' end on the opposite strand (i.e., be classified as Group 5PP, Figure 1B, top), but may also overlap with the coding region of another gene located in its coding region (i.e., be classified as Group EE, Figure 1B, middle). This conflict in classification results in an overestimate of the number of 5PP genes and an underestimate of EE genes, and in part, might explain the similarity of 5PP and EE genes during analyses. Overall, by using our priority scheme, the risk of overestimating gene numbers in each group decreases in the order 5PP, EE, 5PI-EI, COS and CSS.

With these caveats in mind, we suggest that any given random sample of genes likely contains mostly genes that deviate from the stereotypical gene architecture, thereby potentially affecting studies on codon usage bias and substitution rates if the data used are enriched or devoid of genes belonging to particular constellations. For example, the enrichment of datasets with overlapping genes of type 5PP and EE would bias codon usage in an upward direction, whereas substitution rate estimates (Ka/Ks) would be biased downwardly. Thus, even though schemes used to classify genes may be varied, we suggest that our classification provided a reasonable framework to illustrate the fact that gene constellations can affect the functional and evolutionary properties of genes, and thus, attention should be paid to this property.

### Quantification of the constellation effect

Studies continue to reveal genomic and functional properties of genes as correlates of their evolutionary dynamics [1]. Given the increasing number of potentially important biological facets of genes, there is a need to quantify their relative effects on the evolutionary dynamics of genes to enable the identification of those most relevant to genome evolution and those most confounding to evolutionary analyses if ignored [1].

From the results of our multivariate analyses, which were not exhaustive, we deduce that the known effect of recombination rates on codon usage bias was about five times (0.041/0.008; c.f. Table 3) more pronounced than the effect of gene constellation (in terms of the statistical effect of coefficient, R). In addition, during multivariate analysis, the effects of X- versus autosomal linkage and gene function on codon usage bias emerged as greater than one order of magnitude more pronounced than the effect of gene constellation (Table 3). Similarly, in examining Ka/Ks values, we estimated that the expected importance of gene function during multivariate analysis is at least ten times more relevant than the effect of gene constellation (Table 3). We expect that these contrasts between the relative effects of genomic properties that are broadly embraced as important to the evolution of genes and the new property 'gene constellation' examined by us are reasonably informative. This is because these analyses were based on large numbers of genes representing a range of recombination rates (Figure 5, 6, 7, 8).

When analyzing genetic polymorphism data the effect of gene constellation that was observed was absent or subtle (Tables 2, 3). This could be due to limited data availability and/or because the effect truly is weak or nonexistent. Analyses of genetic polymorphism data require immense care and power to distinguish the generally subtle effects of, for example, recombination and demographics. Thus, whether future polymorphisms analyses that are based on more comprehensive samples of genes would be able to uncover subtle effects of gene constellation remains to be seen in our view, and we would expect these to be at least 1-2 orders of magnitudes smaller than the effect of recombination rates.

Thus, despite the fact that the list of potentially interesting evolutionary properties examined was not exhaustive, and the fact that our study should be considered a first-pass analysis of this feature, we showed that gene constellation might factor into some of the functional properties of genes examined (e.g. correlation of expression). However, the effects on evolutionary properties of genes we were able to detect generally were as weak (e.g. codon usage bias, Ka/Ks) or much weaker as we had expected (polymorphism data). We did not correct for multiple testing because our intention was to identify those properties that might be most relevant in this study context. We therefore placed more emphasis on the relative quantification of the effects than on formal significance.

### Implications for hypothesis testing

We examined whether gene constellation might be a genomic property that should be considered more routinely during molecular evolutionary and population genetic studies. Results of multivariate analyses were consistent with a significant influence of gene constellation on a subset of the evolutionary properties studied. However, the effect was weak when expressed in terms of the relative contribution of model features during multivariate model fitting (Table 3). Thus, the practical relevance of ignoring the confounding effects of gene constellation is questionable.

We showed that two data sets were biased in their representation of gene constellations (Table 4). The bias observed is expected to result in a type I error, in that the compositional bias would result in inflated Ka/Ks. This is of concern, because this bias is in the direction of the alternative hypothesis, which in this context posits that male reproductive genes tend to evolve at accelerated rates [2,33].

Results of multivariate analysis showed that gene constellation was important during the study of male reproductive genes. However, the effect did not confound the main conclusion of the study, as the biological function of a gene as a male accessory gland protein was two orders of magnitude more important (in terms of the statistical effect) compared to gene constellation. The authors' examination of Ka/Ks values suggested that such genes evolved rapidly (c.f. [33]), however, unbalanced sampling of gene constellation groups appears to have biased the estimation of Ka/Ks in an upward direction, as predicted from the positive effect of the model feature gene constellation (Table 5). Thus, while the conclusion that male reproductive genes evolve at Ka/Ks above the genomic average remains valid, we would predict that the rates reported for Acp genes are somewhat inflated.

## Conclusion

Application of our classification scheme, or slight variations thereof, shows that the stereotypical solitary gene should be considered an exception in the *Drosophila *genome, rather than the rule. Even when transcriptional territories are deemed irrelevant (e.g., in a particular study context) the solitary gene accounts for only 50% of all genes; indicating that the remaining 50% of genes physically overlap in some way.

The rarity of the solitary gene is worth stating because, firstly, gene constellation has not been discussed in the otherwise complete examination of properties affecting gene function and evolution in Drosophila [1].

Secondly, as we show for the Drosophila genome, some gene constellations deviate in their functional or evolutionary properties from the genome average or from the average of solitary genes.

Moreover, gene constellations emerged as a model feature during multivariate analysis alongside variables known to influence the evolution of genes.

However, the effects were weak, particularly for the evolutionary properties studied, and thus, depending on the biological aspect studied and the types of analyses conducted, this factor appears to be of comparatively small significance or concern. Nevertheless, we suggest that our classification scheme, or some variations thereof, may warrant consideration when results of evolutionary analyses are interpreted.

## Methods

### Classification of gene constellations

#### Solitary genes

Genes with no other adjoining genes on either strand within 20 kb were classified as solitary genes (Group SG) (Figure 1A). At times, we refer to these genes as the stereotypical genes as depicted in textbooks (e.g., [15], Figure 1.4).

#### Overlapping genes

Genes whose 5-prime regions fully or partially overlap with the 5-prime region of another gene were assigned to the group '5-prime overlapping genes' (Group 5PP; Figure 1B, top). The overlap of genes in this group does not result in the overlap of complementary sense and antisense transcripts [24,34,35]. In contrast to previous analyses of gene overlap that considered an overlap distance of 300 bp or less [36], we considered 1,000 bp of non-coding region upstream of the annotated transcription start sites (5-prime region).

We assigned genes to the group of exon-overlapping genes (Group EE; Figure 1B, middle) when the coding regions (exons) fully or partially overlap with the coding regions (exons) of another gene. This type of gene overlap involves the presence of complementary sense and antisense transcripts [24,34,35]. In the field of experimental molecular biology, genes belonging to this group have been termed *cis*-NATs (*cis *natural antisense transcripts) [8,37].

Finally, we assigned genes to the group of intron-overlapping genes (Group 5PI-EI; Figure 1B, bottom) whose 5-prime region or coding region (exons) overlap with the introns of another gene. Except during the brief lifespan of non-processed mRNA, the overlap of genes in this group does not result in the overlap of complementary sense and antisense transcripts. In essence, this type of overlap involves little, if any, direct conflict between functional sites (here: non-synonymous and potentially regulatory sites).

#### Chromatin co-clustering genes

Chromatin co-clustering genes include genes that co-locate within 20 kb of another gene on the opposite strand (Group COS, Figure 1C, top) or on the same strand (CSS, Figure 1C, bottom). In Drosophila, concerted access to chromatin during transcription has been inferred for genes spanning 20 kb segments [11,13], leading to significant correlation in gene expression within co-called transcriptional territories. We varied the distances separating genes to account for the range of distances over which transcriptional territories can occur (2 kb to 200 kb; median 100 kb) (Additional file 2; Figure 2B).

If genes could be assigned to more than one group we applied a priority-ruling scheme: 5PP > EE > 5PI-EI > COS > CSS > SG; where '>' is a placeholder for 'has higher assignment priority than'. See Additional file 1 for the annotation of genes following this scheme.

### *Drosophila *genome sequence data

We downloaded 14,144 transcripts representing unique genes of the *D. melanogaster *genome assembly (Apr 2006) (http://genome.ucsc.edu/cgi-bin/hgTables, accessed via the UCSC Table Browser). We considered the maximum length of known or annotated transcripts, including the maximum length of the coding regions, to account for alternative splicing. We excluded 34 genes that could not be assigned to a group following our classification scheme. These were mostly pairs of genes where the coding region of one gene overlapped with both the 5-prime regions and the coding regions of another gene on the opposite strand. Thus, these could be considered in a broader group, which displays direct conflict between functional sites on opposite strands. However, this group represents too small a sample to be used for statistical comparisons, and the omission of these genes is unlikely to affect our statements.

We generated random datasets through random sampling of genes. The number of randomly drawn genes was chosen to reflect the sample size in each of the gene constellations. The random samples were used as reference points for a number of statistical comparisons (see below).

To examine the conservation pattern of gene constellation between *D. melanogaster *and *D. pseudoobscura*, we obtained 9946 maximum-length transcripts for unique genes of *D. pseudoobscura *genome assembly (Nov 2004) from UCSC database (accessed Dec 2009) and annotated orthologs from the Inparanoid database [27]. Applying a similar classification scheme to the *D. pseudoobscura *genome, we assigned 9941 genes into SG, 5PP, EE, 5PI-EI, COS and CSS constellations. After removing the duplicate genes in *D. melanogaster*, we compared 7546 orthologous gene pairs in both species and observed differential abundance of conserved gene constellation in *D. pseudoobscura *(7%-72%).

### Genomic and functional correlates of gene constellations

#### Chromosomal location

Chromosomal locations of *D. melanogaster *genes were adopted as provided (http://genome.ucsc.edu/cgi-bin/hgTables, accessed Apr 2008).

#### Recombination rates

Regional recombination rates for each gene were obtained from the *D. melanogaster *recombination rate calculator (http://petrov.stanford.edu/cgi-bin/recombination-rates_updateR5.pl, accessed Feb 2009) [38].

#### Gene duplication

The identifiers of 3,459 *D. melanogaster *duplicated genes were obtained from Ensembl (http://www.ensembl.org, accessed Feb 2009).

#### Gene ontology (GO)

GO terms of *D. melanogaster *genes were obtained as a proxy for the function of the gene product. To analyze pairs of genes, four different semantic similarity measures were calculated based separately on each GO term using the Resnik probability function (*Sim*_*Res*_) as described [22,23]. We provide results for *Sim*_*Res *_and used the score with best-matching GO term (*GO*_*BM*_) between genes. We also calculated the Relevance measure, *Sim*_*Rel*_, Lin's measure, *Sim*_*Lin*_, and Jiang and Conrath's measure, *Sim*_*JC*_. Moreover, we calculated the alternative measures for *GO*_*BM*_, namely maximum score GO_max _and average score GO_avg _as described [22,23]. For genes for which all three GO term annotations (BP, MF, and CC) were available, the combined functional similarity scores *All*_*max *_was calculated as described [22].

In the main text we refer only to results of the analysis of *GO*_*BM*_. We note that results of analyses based on the other functional similarity scores (*GO*_*max *_and *GO*_*avg*_) and semantic measures (*Sim*_*JC*_, *Sim*_*Lin*_, *Sim*_*Res*_) were in agreement with the analysis of *Sim*_*Res *_and *GO*_*BM *_(not shown).

ProfCom (http://webclu.bio.wzw.tum.de/profcom/, accessed Apr 2009) was used to depict the functional enrichment of GO terms for each gene constellation of *D. melanogaster *genes (c.f. Figure 1) as a heat map [39].

#### **Gene expression**

To analyze the correlation of expression of *D. melanogaster *genes, normalized expression levels of 13,165 transcripts (representing 13,141 unique genes) were obtained http://jbiol.com/content/supplementary/1475-4924-1-5-S1.txt[11]. These data, collected using the Affymetrix microarray platform, cover 6 investigations and 89 experimental conditions imposed on embryo and adult flies at various time points during development. The Pearson correlation coefficient, R, was calculated across conditions and time points. We considered only those genes for which the number of data points were equal or greater than 20 and R was significant at P-value ≤ 0.05. Similarly, as suggested [40,41], absolute values of R ≥ 0.6 were taken as a threshold to detect significant co-expression. An analysis that considered all R-values (i.e., when no threshold for R was applied) or an analysis with higher thresholds led to similar conclusions (data not shown). For statistical comparison we randomly paired 2,000 genes drawn from each gene constellation.

### Evolutionary correlates of gene constellations

For a summary of sample sizes underlying the following analyses c.f. Additional file 4.

#### Codon usage in *D. melanogaster*

The Codon Adaptation Index (CAI) was calculated for *D. melanogaster *genes using the software CodonW http://codonw.sourceforge.net/. CAI measures the synonymous codon usage bias for a DNA or RNA sequence [42]. The measurement ranges between 0 and 1, with a value of 1 indicating extreme codon usage bias.

#### DNA sequence divergence

*D. melanogaster *and *D. pseudoobscura *orthologs were downloaded from the Inparanoid database (http://inparanoid.sbc.su.se/cgi-bin/e.cgi, accessed Jul 2008). These were matched to our *D. melanogaster *set of genes and our gene constellations. The coding and amino acid sequences (CDS) were retrieved via batch download from FlyBase (http://flybase.org/, accessed Jul 2008) and pairwise sequence alignments between *D. melanogaster *and *D. pseudoobscura *gene orthologs were conducted in ClustalW 2.0.9 using the default settings [43]. The rates of synonymous substitutions per synonymous site, Ks, and nonsynonymous substitutions per non-synonymous site, Ka, as well as the resulting Ka/Ks ratio were estimated using the maximum likelihood model of sequence evolution as implemented in the PAML software [44]. *D. pseudoobscura *was chosen as the species for comparative analyses because the evolutionary distance separating it from *D. melanogaster *enables robust estimations of Ka and Ks. Moreover, orthologs are comparatively well agreed upon and available from Inparanoid.

#### DNA sequence polymorphism

We downloaded polymorphism data of *D. melanogaster *genes for 10 African and 40 North American strains covering the X- and the 2^nd ^chromosome from the *Drosophila *Population Genomics Project (DPGP) (http://www.dpgp.org/melanogaster/, accessed Feb 2009). π (θ_π_) and θ_W_, as well as Tajima's D were used to measure genetic variation, and differentiation between African and American flies was measured as a measurement of F_ST_. Calculations were done as implemented in the SITES software [45]. Note that the dataset is biased towards high recombination rates (c.f. main text, Figure 5, 6, 7, 8).

#### Gene expression divergence

Gene expression data were obtained from Gene Expression Omnibus (GEO, http://www.ncbi.nlm.nih.gov/geo/, series accession GSE2642, accessed Apr 2009) [32,46]. The expression values of genes measured for four strains of *D. melanogaster *and one strain of *D. yakuba *(eight replicates per strain) were extracted and averaged to generate a non-redundant dataset using the Bioconductor module in R [47]. Applying the threshold of percentage of false prediction (pfp) ≤ 0.05, 648 genes were identified as being differentially expressed between *D. melanogaster *and *D. yakuba*.

#### Gene expression polymorphism

Gene expression data of 16 *D. melanogaster *strains from African and European populations were obtained [48], with estimated relative expression levels using BAGEL (Bayesian Analysis of Gene Expression Levels) [49]. Gene expression levels were used to measure quantitative trait differences between populations, Q_ST_, which we took as a measure of the differences in gene expression between populations [50].

### Quantification of the influence of gene constellation on evolutionary correlates of genes

For the quantification analysis, in addition to gene constellation, we chose to investigate the effect of recombination rate and gene function on evolutionary correlates (while accounting for the X- versus autosomal linkage). While there are a number of other such potentially important properties, we focused on these because their importance to the evolutionary dynamics of genes is comparatively well understood and quantified. Moreover, we readily recovered the importance of both properties during our analyses, and thus, were able to evaluate the importance of gene constellation in relative terms (see Results). However, the analysis of population genetic variables was limited by data availability (Additional file 4) and a bias towards high recombination rates available for study.

Specifically, we examined the relationship between recombination rate and other evolutionary correlates after filtering out the effect of GO terms that were overrepresented in each gene constellation (Figures 5, 6, 7).

To compare the effect of gene constellation with the better-known effect of recombination rate and X- versus autosomal linkage, we build a parsimonious model using stepwise regression analysis [30]. Data were first standardized into normally distributed z-scores. The best fitting models were selected after stepwise regression analysis, i.e., those with significant properties and coefficients that account for functional or genomic properties. During regression analysis, genes with overrepresented GO terms were encoded as 1 versus the remaining genes that were encoded as 0. Similarly, genes located on the X chromosome were encoded as 1 and genes located on autosomes were numbered as 0.

### Case study datasets

Genes that are involved in reproduction, sexual selection and sexual conflict have become a paradigm for the role of selection in molecular evolution. High evolutionary rates, as measured by Ka/Ks values, are the salient feature of datasets comprised of such genes [32,33,51]. However, high Ka/Ks could also be explained by a bias in the representation of gene constellations in such datasets (see Results). To investigate whether such a bias can be detected in these datasets, we chose one dataset that comprehensively described the sex-biased expression of Drosophila genes [32]. Genes (N = 748) in the sex biased expression dataset matched up with the genes that we classified according to the scheme depicted in Figure 1. Of these 748 genes, 161 genes were categorized as male-bias expressed genes [25]. We performed a Chi-square test to test the null hypothesis that the distribution of genes was unbiased, i.e., that gene constellations were represented in the male-biased set of genes as expected based on their abundances in the genome.

A second dataset comprised the male accessory gland protein (Acp) genes [33] and the molecular evolutionary substitution rates Ka, Ks, and Ka/Ks calculated between *D. melanogaster *and *D. simulans*. We were able to match 50 of these genes to our data without ambiguity. Two-sample proportional tests were utilized to identify over- and under- representation of Acp genes in each gene constellation.

## Competing interests

The authors declare that they have no competing interests.

## Authors' contributions

MHK conceived and directed the study together with the co-authors SL and CS, who also performed the programming and statistical analyses. MHK wrote the manuscript with significant help by SL and CS. All the authors approved the final manuscript.

## Supplementary Material

Additional file 1 **Gene list for each constellation**. Gene list for each constellation List of genes and their classification as solitary, paired overlapping, and chromatin co-clustering genes following our scheme depicted in Figure 1. Genomic, functional, and evolutionary properties obtained/calculated used for analyses are provided for each gene. Click here for file

Additional file 2 **Gene Ontology analysis**. List of Gene Ontology terms and overrepresented gene constellations in functional enrichment analysis. Click here for file

Additional file 3 **The conservation of gene constellations between *D. melanogaster *and *D. pseudoobscura*, with (Additional file **3A)** and without (Additional file **3B**) duplicate genes.** Click here for file

Additional file 4 **The number of genes in each constellation**. Sample sizes underlying analysis of functional and evolutionary properties of genes (c.f. Table 2). Click here for file

Additional file 5 **Figures of evolutionary properties relative to recombination rates for gene constellations. (Additional file **5A** i.-v.) Prior to the elimination of overrepresented GO terms for the X-chromosome. (Additional file **5B** i.-v.) Prior to the elimination of overrepresented GO terms for autosomes.** Click here for file
