# Supplementary material for: Functional and evolutionary correlates of gene constellations in the Drosophila melanogaster genome that deviate from the stereotypical gene architecture
Source: BMC Genomics. 2010 May 24;11:322. doi: 10.1186/1471-2164-11-322 (PMC2891614; doi:10.1186/1471-2164-11-322)
Supplement: Additional file 4 — The number of genes in each constellation. Sample sizes underlying analysis of functional and evolutionary properties of genes (c.f. Table 2). [file 1471-2164-11-322-S4.DOC]

**Additional file 4. The number of genes in each constellation^a^.** Sample sizes underlying analysis of functional and evolutionary properties of genes (c.f. Table 2).

| **Group** | **Recombination rate** | **CAI** | **Ka/Ks** | **African population** | | | **American population** | | | | **F_ST_** | **Expr-Div^c^** | **Q_ST_** |
| --- | --- | --- | --- | --- | --- | --- | --- | --- | --- | --- | --- | --- | --- |
| **θ_ω_** | **θ_π_** | **Tajima’s D** | **θ_ω_** | **θ_π_** | **Tajima’s D** | |
| **Genes on the autosomes** | | | | | | | | | | | | |  |
| **SG** | 624 | 596 | 351 | 29 | 29 | 29 | 29 | 29 | | 29 | 29 | 17 of 624 | 180 |
| **5PP** | 3491 | 3419 | 2797 | 87 | 87 | 87 | 87 | 87 | | 87 | 87 | 117 of 3491 | 1143 |
| **EE** | 629 | 618 | 486 | 7 | 7 | 7 | 7 | 7 | | 7 | 7 | 17 of 629 | 179 |
| **5PI-EI** | 531 | 507 | 307 | 28 | 28 | 28 | 28 | 28 | | 28 | 28 | 33 of 531 | 143 |
| **COS** | 2932 | 2796 | 2001 | 94 | 94 | 94 | 94 | 94 | | 94 | 94 | 123 of 2932 | 846 |
| **CSS** | 565 | 527 | 347 | 15 | 15 | 15 | 15 | 15 | | 15 | 15 | 33 of 565 | 160 |
| **Genomic average^a^** | 8772 | 8463 | 6289 | 260 | 260 | 260 | 260 | 260 | | 260 | 260 | 340 of 8772 | 2651 |
| **Gene on the X chromosome** | | | | | | | | | | | | |  |
| **SG** | 153 | 142 | 69 | 24 | 24 | 24 | 24 | 24 | | 24 | 24 | 1 of 155 | 49 |
| **5PP** | 670 | 652 | 510 | 78 | 78 | 78 | 78 | 78 | | 78 | 78 | 14 of 671 | 227 |
| **EE** | 94 | 91 | 74 | 7 | 7 | 7 | 7 | 7 | | 7 | 7 | 1 of 94 | 27 |
| **5PI-EI** | 104 | 99 | 62 | 5 | 5 | 5 | 5 | 5 | | 5 | 5 | 5 of 104 | 38 |
| **COS** | 582 | 551 | 343 | 63 | 63 | 63 | 63 | 63 | | 63 | 63 | 17 of 582 | 196 |
| **CSS** | 142 | 123 | 78 | 24 | 24 | 24 | 24 | 24 | | 24 | 24 | 5 of 142 | 50 |
| **Genomic average^b^** | 1745 | 1658 | 1136 | 201 | 201 | 201 | 201 | 201 | | 201 | 201 | 43 of 1748 | 587 |

**^a^** c.f. Additional File 1 for data.

**^b^**Average values except for the measure for expression divergence (Expr-Div), where the value provided should be considered the genome wide proportion of differentially expressed genes.

**^c^** Expression Divergence: the number of differentially expressed genes between *D. melanogaster* and *D. yakuba* of total gene number in each group*.*
