# Supplementary material for: Functional and evolutionary correlates of gene constellations in the Drosophila melanogaster genome that deviate from the stereotypical gene architecture
Source: BMC Genomics. 2010 May 24;11:322. doi: 10.1186/1471-2164-11-322 (PMC2891614; doi:10.1186/1471-2164-11-322)
Supplement: Additional file 5 — Figures of evolutionary properties relative to recombination rates for gene constellations. (Additional file 5A i.-v.) Prior to the elimination of overrepresented GO terms for the X-chromosome. (Additional file 5B i.-v.) Prior to the elimination of overrepresented GO terms for autosomes. [file 1471-2164-11-322-S5.DOC]

**Additional file 5. Evolutionary properties relative to recombination rates for each gene constellation. (A) Prior to the elimination of overrepresented GO terms for the X-chromosome. (B) Prior to the elimination of overrepresented GO terms for autosomes.**

**A**. **Evolutionary properties and recombination rates of genes on X chromosome in different constellations (prior to the elimination of overrepresented GO terms). (i)** Codon usage bias predicator CAI (Codon Adaptation Index); sample size for each group: SG (142), 5PP (652), EE (91), 5PI-EI (99), COS (551), CSS (123). **(ii)** The ratio of fixation of amino-acid replacement mutations (Ka) over the rate of synonymous mutations (Ks) between *D. melanogaster* and *D. pseudoobscura*; sample size for each group: SG (69), 5PP (510), EE (74), 5PI-EI (62), COS (343), CSS (78). **(iii)** African population q_w_ q_p_, Tajima’s D; sample size for each group: SG (24), 5PP (78), EE (7), 5PI-EI (5), COS (63), CSS (24). **(iv)** Sequence differentiation among populations F_ST_; sample size for each group: SG (24), 5PP (78), EE (7), 5PI-EI (5), COS (63), CSS (24). **(v)** Gene expression differentiation among populations Q_ST_; sample size for each group: SG (49), 5PP (227), EE (27), 5PI-EI (38), COS (196), CSS (50). Bars represent 95% confidence interval. Compared to solitary genes, * indicate significant difference of recombination rate (X-axis) at *P* < 0.05, + indicate significant difference of other evolutionary properties (Y-axis) at *P* < 0.05 (Mann-Whitney test, two-sided, not corrected for multiple testing).

**i.**

**
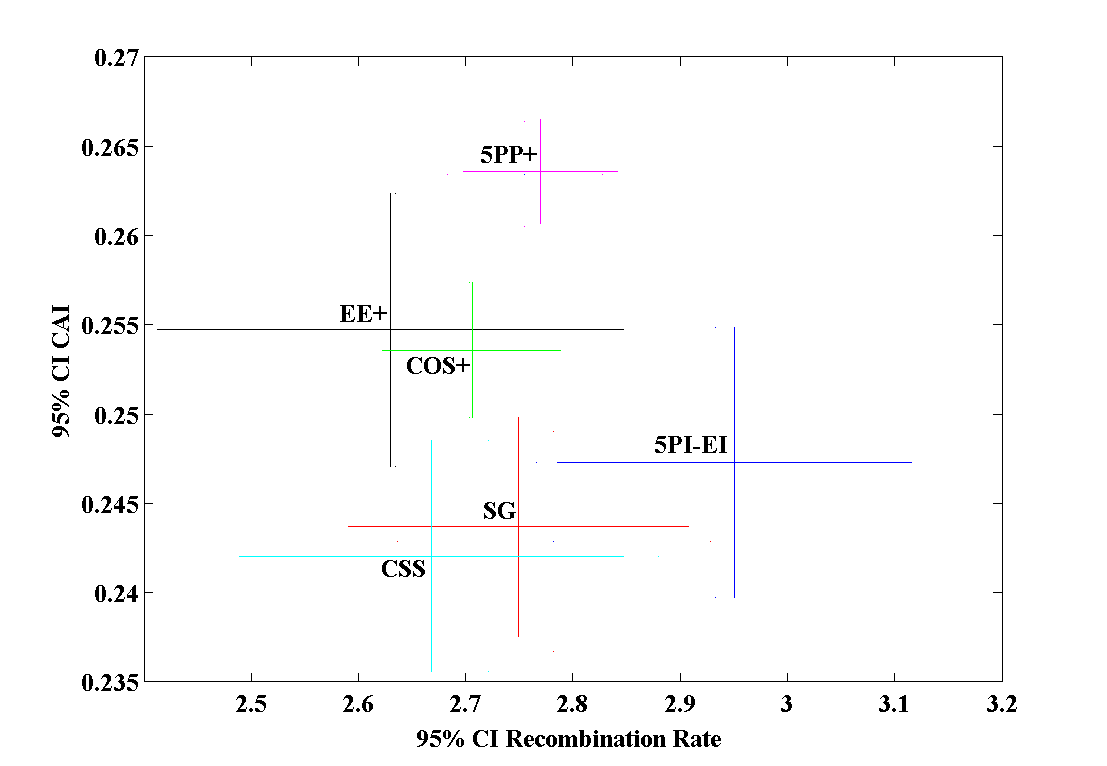
**


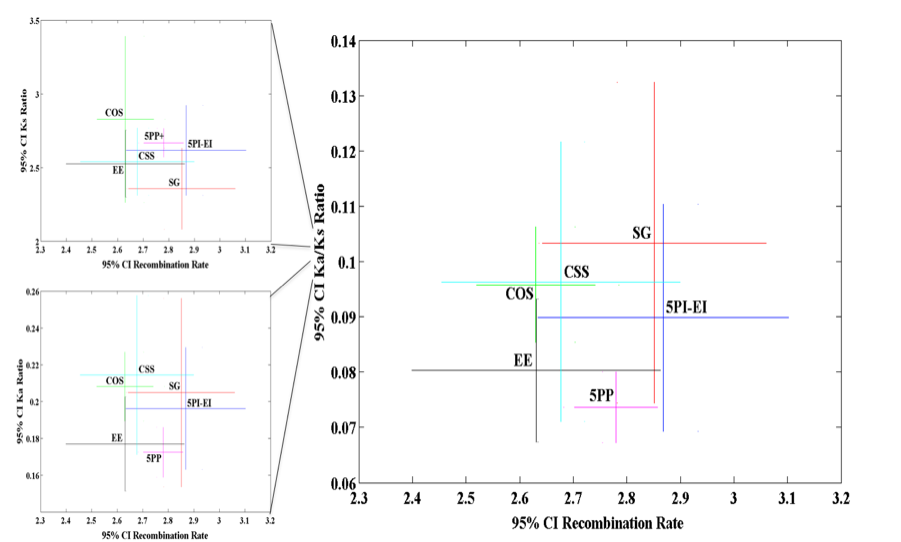
**ii.**


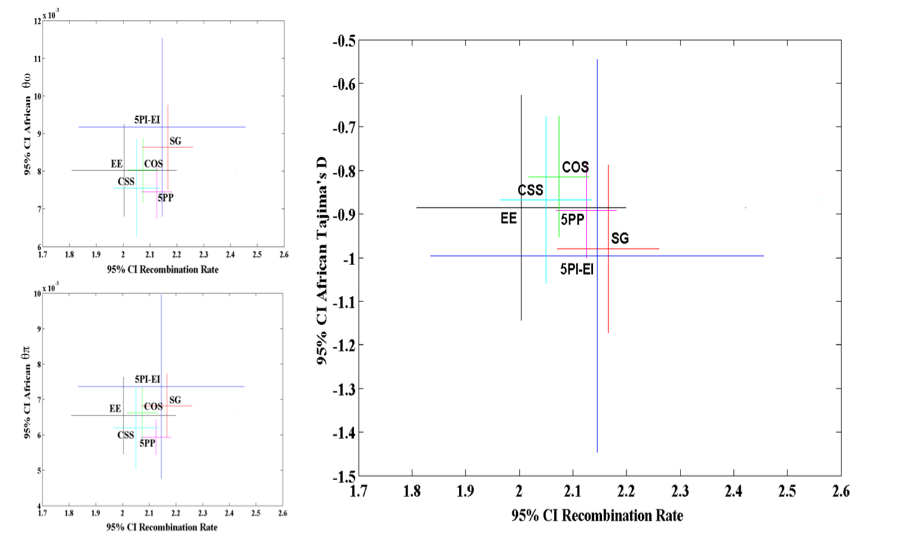
**iii.**


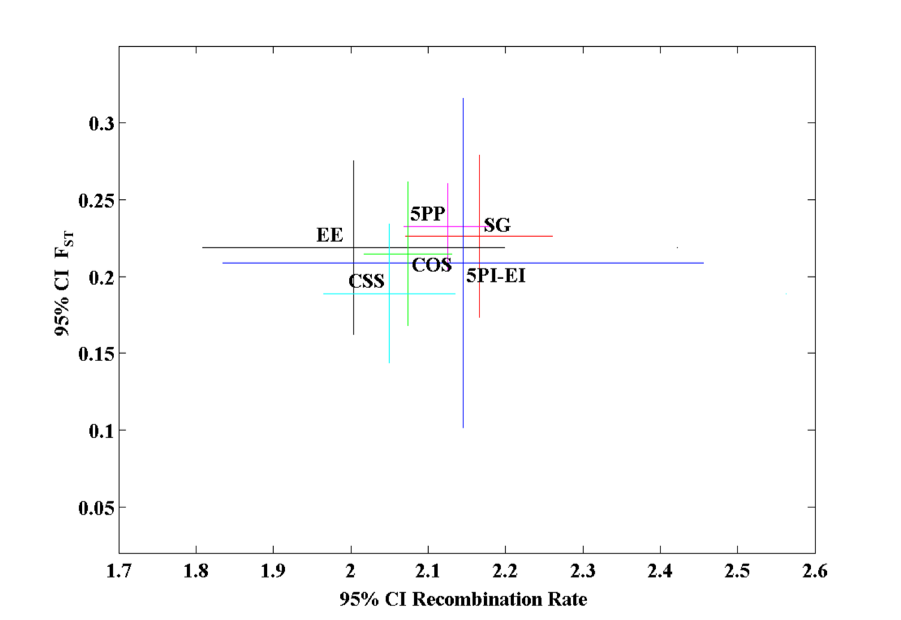
**iv.**


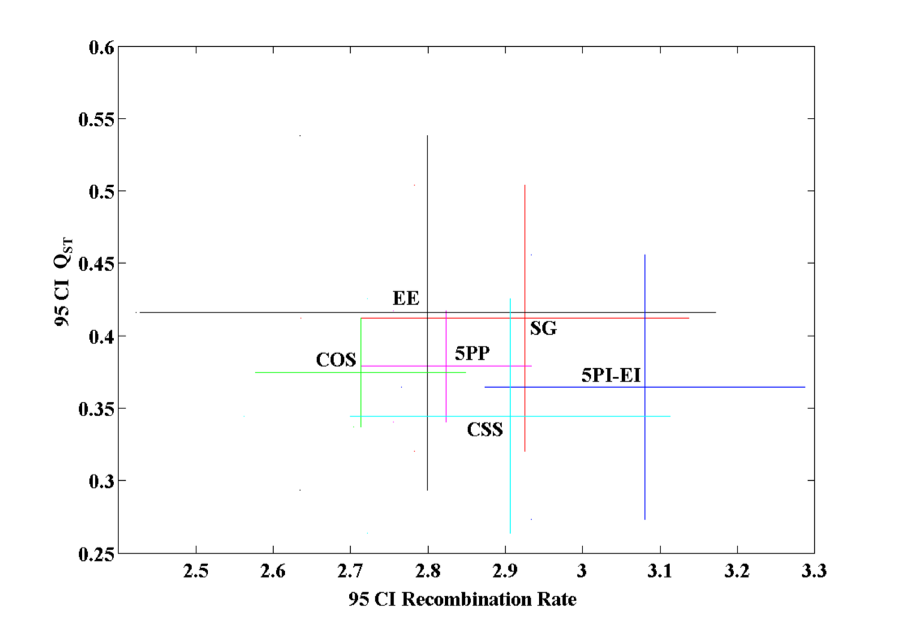
**v.**

**B**. **Evolutionary properties and recombination rates of genes on autosomes in different constellations (prior to the elimination of overrepresented GO terms). (i)** Codon usage bias predicator CAI (Codon Adaptation Index); sample size for each group: SG (596), 5PP (3419), EE (618), 5PI-EI (507), COS (2796), CSS (527). **(ii)** The ratio of fixation of amino-acid replacement mutations (Ka) over the rate of synonymous mutations (Ks) between *D. melanogaster* and *D. pseudoobscura*; sample size for each group: SG (351), 5PP (2797), EE (486), 5PI-EI (307), COS (2001), CSS (347). **(iii)** African population q_w_ q_p_, Tajima’s D; sample size for each group: SG (29), 5PP (87), EE (7), 5PI-EI (28), COS (94), CSS (15). **(iv)** Sequence differentiation among populations F_ST_; sample size for each group: SG (29), 5PP (87), EE (7), 5PI-EI (28), COS (94), CSS (15). **(v)** Gene expression differentiation among populations Q_ST_; sample size for each group: SG (180), 5PP (1143), EE (179), 5PI-EI (143), COS (846), CSS (160). Bars represent 95% confidence interval. Compared to solitary genes, * indicate significant difference of recombination rate (X-axis) at *P* < 0.05, + indicate significant difference of other evolutionary properties (Y-axis) at *P* < 0.05 (Mann-Whitney test, two-sided, not corrected for multiple testing).

**i.
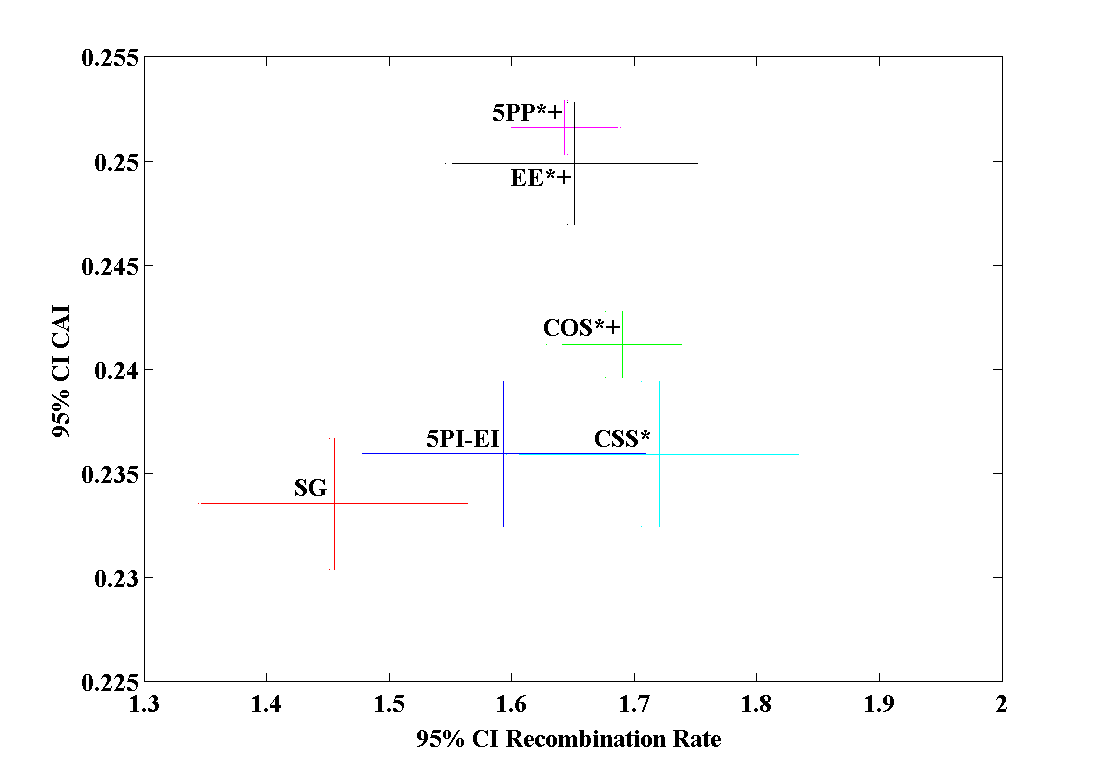
**


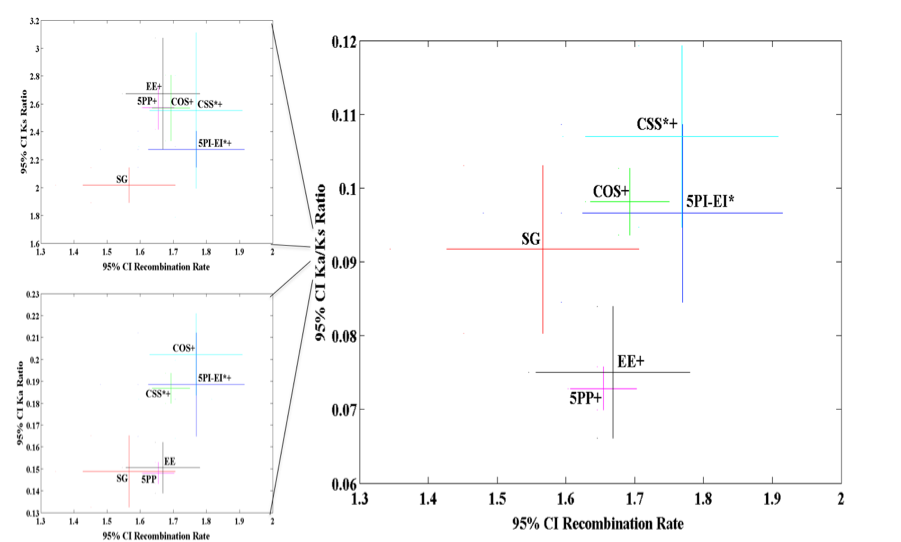
**ii.**


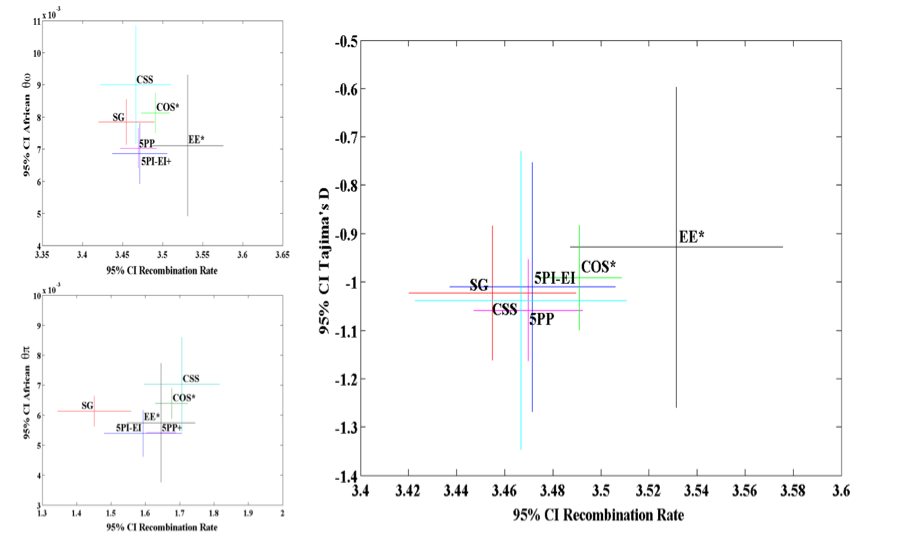
**iii.**

**iv.**


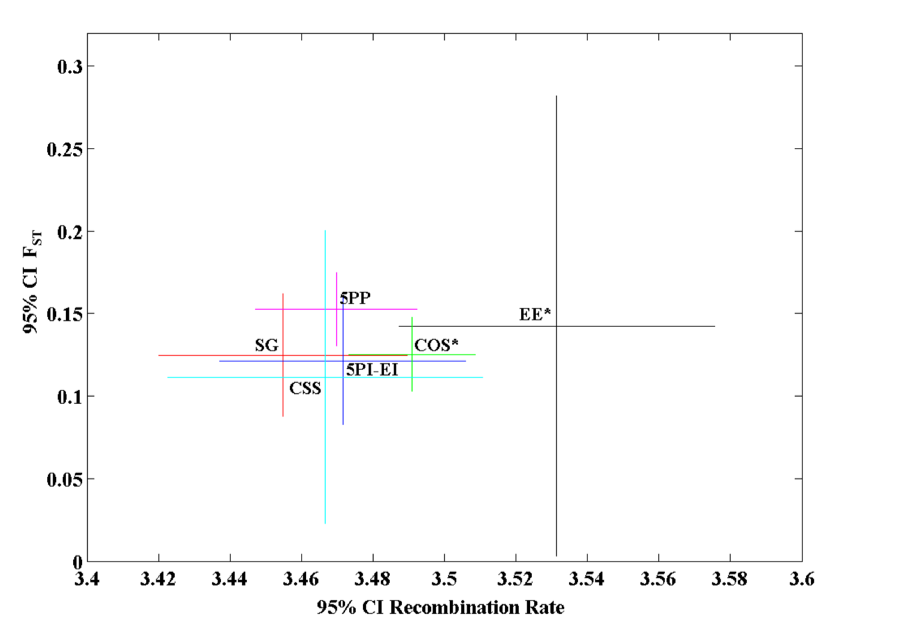

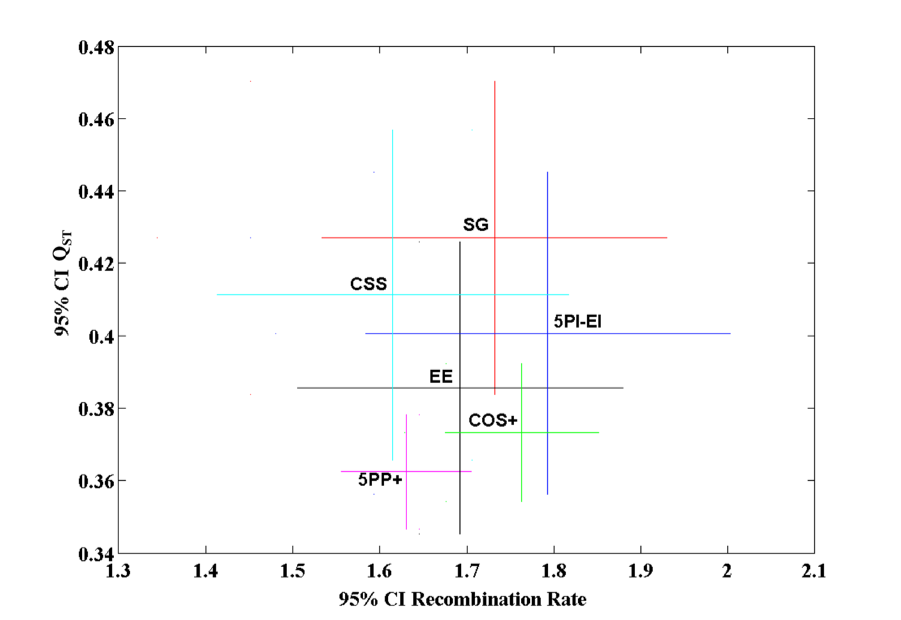
**v.**
